# Aberrant DNA methylation of the p16^INK4a^ gene in plasma DNA of breast cancer patients

**DOI:** 10.1038/sj.bjc.6690495

**Published:** 1999-06

**Authors:** J M Silva, G Dominguez, M J Villanueva, R Gonzalez, J M Garcia, C Corbacho, M Provencio, P España, F Bonilla

**Affiliations:** Department of Medical Oncology, Clinica Puerta de Hierro, Madrid, 28035, Spain

**Keywords:** p16^INK4a^ gene, DNA methylation, breast cancer, plasma DNA, CpG islands

## Abstract

Hypermethylation of exon 1 of p16^INK4a^ was examined in tumour and plasma DNA of a series of breast cancer patients. De novo methylation was observed in the tumours of eight patients (23%), and in plasma DNA in five (14%) of these eight patients. Our data show that de novo methylation of exon 1 of p16^INK4a^ can be demonstrated in plasma DNA of breast cancer patients, a fact that provides additional evidence of the tumour-related origin of free plasma DNA in cancer patients. © 1999 Cancer Research Campaign


*British Journal of Cancer* (1999) **80**(8), 1262–1264
© 1999 Cancer Research Campaign
Article no. bjoc.1999.0495

### Aberrant DNA methylation of the p16$^{INK4a}$ gene in plasma DNA of breast cancer patients

**JM Silva, G Dominguez, MJ Villanueva, R Gonzalez, JM Garcia, C Corbacho, M Provencio, P España and F Bonilla**

Department of Medical Oncology, Clinica Puerta de Hierro, 28035-Madrid, Spain

**Summary** Hypermethylation of exon 1 of p16$^{INK4a}$ was examined in tumour and plasma DNA of a series of breast cancer patients. De novo methylation was observed in the tumours of eight patients (23%), and in plasma DNA in five (14%) of these eight patients. Our data show that de novo methylation of exon 1 of p16$^{INK4a}$ can be demonstrated in plasma DNA of breast cancer patients, a fact that provides additional evidence of the tumour-related origin of free plasma DNA in cancer patients.

**Keywords**: p16$^{INK4a}$ gene; DNA methylation; breast cancer; plasma DNA; CpG islands

The p16$^{INK4a}$ gene encodes a protein, p16, which is a D-type cyclin-dependent kinase (cdk) inhibitor that blocks the ability of cdk4 to interact with cyclin $D_1$ and stimulate the progression of eukaryotic cells through G1 phase of the cell cycle (Serrano et al, 1993). The p16$^{INK4a}$ gene was found to be inactivated in a large percentage of tumour cell lines (Kamb et al, 1994; Nobori et al, 1994; Okamoto et al, 1994). However, its inactivation is observed less frequently in primary tumours than in the cell lines (Serrano, 1997). There are three main mechanisms of genetic inactivation of p16$^{INK4a}$: deletions of both alleles, deletion of one allele and intragenic mutation of the remaining allele; and deletion of one allele and methylation-mediated silencing of the remaining allele (Serrano, 1997).

Gene silencing has been associated with methylation of a CpG island located in, or near, promoters and 5′ regulatory regions (Herman et al, 1994; Issa et al, 1994). CpG islands are G+C rich regions that show a higher frequency of CpG dinucleotides than is normally seen in the vertebrate genome, that are not methylated in the germline and, with some exceptions, are usually unmethylated in normal somatic cells (Bird, 1986). In contrast, widespread methylation of CpG islands occurs on autosomal genes during the carcinogenic process (Jones and Buckley, 1990; Laird and Jaenisch, 1994). The exon 1 coding sequences of the p16$^{INK4a}$ gene reside within 5′ CpG islands (Gonzalez-Zulueta et al, 1995), and transcriptional block of p16$^{INK4a}$ by methylation of its 5′ CpG island has been reported in many cancer cell lines (Gonzalez-Zulueta et al, 1995; Herman et al, 1995; Merlo et al, 1995; Poster et al, 1998), as well as in primary tumours (Gonzalez-Zulueta et al, 1995; Herman et al, 1995; Merlo et al, 1995). Among them, breast cancer displayed a rate of 31% of de novo methylation (Herman et al, 1995).

For more than two decades scientists have known that minute quantities of DNA are present in the plasma of normal individuals, and that increased quantities circulate in patients with chronic autoimmune disorders and cancer (Leon et al, 1977; Shapiro et al, 1983). The source of this circulating DNA remains enigmatic. A certain amount seems to originate from lymphocytes (Anker et al, 1975), but in cancer patients, a tumour-related origin cannot be ruled out (Stroun et al, 1987, 1989), as these individuals show greater levels of DNA than healthy subjects. Moreover, these levels correlate inversely with outcome and tend to fall with effective treatment (Leon et al, 1977). More recent studies have involved the use of polymerase chain reaction (PCR) to amplify plasma DNA and identify genetic alterations in it that correspond to the same alterations found in tumour DNA. The sensitivity of PCR techniques in the detection of these alterations in plasma DNA may be as high as 86% (Anker et al, 1997); and the specificity shown in the studies in this field ranged from about 28% to 100% (Anker et al, 1997; Mulcahy et al, 1998; Sanchez-Cespedes et al, 1998). Among these alterations, K-*ras*, N-*ras* and p53 gene mutations have been reported in the plasma DNA of patients with pancreatic cancer, colorectal cancer and acute myelogenous leukaemia (Vasioukhin et al, 1994; Anker et al, 1997; Hibi et al, 1998). Likewise, specific microsatellite alterations have been detected in patients with small-cell lung cancer (SCLC) (Chen et al, 1996), non-SCLC (Sanchez-Cespedes et al, 1998), head and neck carcinomas (Nawroz et al, 1996) and clear cell renal carcinoma (Goessl et al, 1998).

In this context, we investigated whether one of the inactivation mechanisms of p16$^{INK4a}$, hypermethylation, was present in our patients with breast carcinomas and the possibility of finding this phenomenon in plasma DNA of these patients.

### MATERIALS AND METHODS

### Tissue samples and DNA extraction

All participants were informed of the nature of the study, and gave their informed consent. A tumour sample was obtained at surgery from each of 35 patients with breast cancer, immediately after resection, and snap-frozen in liquid nitrogen until processing. All specimens underwent histological examination to confirm the

*Received 10 September 1998*
*Revised 21 December 1998*
*Accepted 12 January 1999*

*Correspondence to:* F Bonilla, Molecular Genetics Unit, Clinica del Trabajo, Avd. de la Reina Victoria 21, 28003-Madrid, Spain diagnosis of invasive breast carcinoma. Pathological diagnosis and clinical evaluation disclosed no evidence of metastatic dissemination in any patient. A blood sample was collected from each patient before surgery. DNA extraction of tumour samples and of peripheral mononuclear cells (used as normal DNA to avoid possible molecular alterations of normal breast tissue) was performed by a non-organic method (Oncor Inc, Gaithersburg, MD, USA). Plasma DNA was purified on Qiagen columns (Qiamp Blood Kit; Qiagen Inc., Hilden, Germany) according to the blood and body fluids protocol, introducing the following modifications. Between 7.5 and 12 ml of plasma were heated at 99°C for 5 min on a heat block. The heated sample was then centrifuged at 14 000 rpm for 30 min, after which the clear supernatant was collected (Lo et al, 1997). Proteinase K (20 mg ml$^{-1}$) (Boehringer Mannheim, Mannheim, Germany) and buffer AL (Qiagen Inc.) were added in a 1/10 proportion with respect to the collected supernatant and incubated overnight at 55°C.

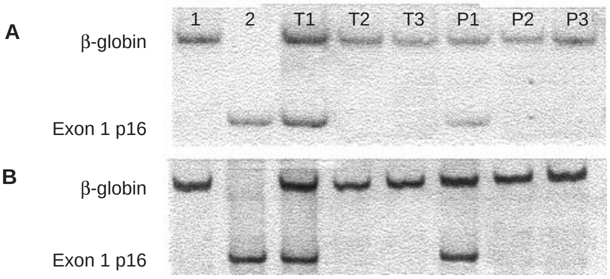

**Figure 1** Photograph of the gels showing the PCR-based methylation analysis of exon 1 of the p16$^{INK4a}$ gene in samples of tumour (T) and plasma (P) DNA. (**A**) Results after DNA digestion with *Sa*$^{eII}$ restriction enzyme, (**B**) results for the same cases after digestion with *P*$^{stI}$ restriction enzyme. The different lanes are as follows: lane 1, β-globin alone in a control case; lane 2, exon 1 of p16$^{INK4a}$ isolated in a control; lanes $T_1$ and $P_1$ (patient no. 1), amplification of exon 1 of p16$^{INK4a}$ after digestion with restriction enzyme *Sa*$^{eII}$ in tumour DNA ($T_1$) and plasma DNA ($P_1$), indicating presence of methylation; lanes $T_2$, $T_3$ and $P_2$, $P_3$, corresponding to patient no. 2 and 3, no amplification of exon 1 of p16$^{INK4a}$ was present after digestion, suggesting absence of DNA methylation

### PCR-based methylation assay

A PCR assay, based on the inability of some restriction enzymes to cut methylated sequences as described (Singer-Sam et al, 1990), was used to analyse the methylation status of the first exon of p16$^{INK4a}$, and the *Pst*I and *Sac*II sites were examined. Analysis of DNA digests was performed according to the manufacturer's directions (Promega, Madison, WI, USA). DNA (250 ng) was digested overnight with 2.5 units of enzyme; 100 ng of the digested DNA were amplified with primers flanking the restriction sites. Amplification of β-globin, adding the corresponding primers, was used as internal control of the reaction in a multiplex PCR. (There are no restriction sites in the β-globin sequence selected for *Pst*I and *Sac*II). The primer set used for methylation analysis of exon 1 of p16$^{INK4a}$ was 5′-GGG AGC AGC ATG GAG CCG-3′ (sense) and 5′-AGT CGC CCG CCA TCC CCT-3′ (antisense); and for β-globin, 5′-CAA CTT CAT CCA CGT TCA CC-3′ (sense) and 5′-GAA GAG CCA AGG ACA GGT AC-3′ (antisense). The conditions were as follows: buffer 10X 2.5 μl, dNTPs 200 μM, $Cl_2Mg$ 2.5 mM, exon 1 primers 0.6 μM, β-globin primers 0.6 μM and 100 ng of the digested DNA as template and $dH_2O$ to reach a total volume of 25 μl. This reaction mixture was amplified with Ampli *Taq* Gold (Perkin Elmer, Roche Molecular Systems Inc., Branchburg, NJ, USA): 94°C for 12 min, 35 cycles of 94°C for 30 s, 61°C for 30 s and 72°C for 30 s, followed by incubation at 72°C for 11 min. PCR products were resolved in 6% acrylamide and stained with a non-isotopic silver nitrate method (Oto et al, 1993). PCR-based methylation analysis using restriction enzymes may be subject to variability if the DNA digestions are not complete. To rule out the possibility of incomplete restriction, all samples were digested overnight, and twice in independent experiments. PCR amplifications from each of the duplicate digests were repeated twice to ensure reproducibility of the results.

### RESULTS

The methylation status of two CpG sites (*Pst*I and *Sac*II) within the first exon of p16$^{INK4a}$ was examined in tumour and normal DNA of 35 patients with breast cancer. The sites were analysed by a PCR-based assay using methylation-sensitive enzymes for DNA cleavage, followed by PCR amplification of the digested DNA, utilizing the aforementioned primers of the first exon of p16$^{INK4a}$. The analysis of normal DNA displayed no methylation of the exon studied because no PCR product was generated following digestion with the restriction enzyme used. After cutting with the endonucleases, eight tumours (23%) showed de novo methylation of exon 1 of p16$^{INK4a}$, since PCR products were generated after digestion. In all of these cases, methylation was observed after digestion with *Pst*I and *Sac*II restriction enzymes. In the three cases in which the second amplification of each of the duplicate digested samples displayed discordant results, a third, decisive amplification was performed. Plasma DNA extraction disclosed concentrations ranging from 20 ng ml$^{-1}$ to 178 ng ml$^{-1}$, with an average of 110 ng ml$^{-1}$ among our 35 patients, none of whom presented metastases. Seventeen healthy controls were also analysed. Their plasma DNA concentrations were lower than those of cancer patients, ranging between 0 and 45 ng ml$^{-1}$. In all cases, the DNA extracted was suitable for molecular study; in order to test it, exon 1 of p16$^{INK4a}$ was amplified prior to the PCR-based methylation assay, and the products electrophoresed in 1.5% agarose were similar to the products obtained with control DNA. Next, plasma DNA was analysed for presence of methylation, using the same PCR-based methylation assay performed in tumour DNA. In five (14%) of the eight patients that showed de novo methylation in tumour DNA, this phenomenon was demonstrated in plasma DNA (Figure 1). Plasma DNA methylation was not observed in any of the 27 cases in which there was no methylation in tumour DNA.

### DISCUSSION

Exon 1 of p16$^{INK4a}$ is contained in a CpG island that has a density of CpG of 8.6%, which exceeds the theoretically expected frequency of CpG dinucleotides (Bird, 1986; Gardiner-Garden and Frommer, 1987; Merlo et al, 1995) in mammalian DNA. These islands are unmethylated in normal tissue (Merlo et al, 1995) although, exceptionally, the absence of p16$^{INK4a}$ expression in association with methylation of its 5′ CpG could be observed in normal colon mucosa (Gonzalez-Zulueta et al, 1995). The present rate, 23%, of de novo methylation found in primary tumours of our patients with breast cancer is lower than the 31% reported by Herman et al (1995). The number of tumours checked and restriction enzymes used may influence this result.

© 1999 Cancer Research Campaign

In studies reporting the same type of molecular changes in both tumour DNA and plasma DNA (Vasioukhin et al, 1994; Chen et al, 1996; Nawroz et al, 1996; Anker et al, 1997; Hibi et al, 1998; Sanchez-Cespedes et al, 1998), molecular markers have been selected that display a high rate of specific alterations. However, to our knowledge, exon 1 of p16$^{INK4a}$ had not been utilized to date to check plasma DNA in breast cancer patients, and there are no available data concerning its informativeness. We found 14% of de novo methylation in plasma DNA of our patients, a fact that demonstrates that the existence of plasma DNA in patients harbouring a breast carcinoma is a tangible observation. The finding that the aberrant DNA methylation in exon 1 of p16$^{INK4a}$ in plasma DNA of these patients is identical to the alteration present in the corresponding carcinomas indicates that hypermethylated plasma DNA derives from the primary tumour. However, the origin of plasma DNA remains speculative; thus, lymphocytes and other molecular mononuclear blood cells may be a direct source of plasma DNA, as may cells of solid tissues with a high turnover rate such as tumour cells (Stroun et al, 1989). In this context, the exhibition of the molecular change in tumour DNA but not in plasma DNA, as occurred in three of our patients, may be the result of the blocking of the incorporation of free tumour DNA from tumour cells into the blood, or a possible increase in the rate of the plasma DNA degradation process mediated by nucleases.

### ACKNOWLEDGEMENTS

This work was supported by grants from the Fundacion Caja Madrid and FIS no. 98/0847. We are grateful to the patients who participated in this study and to Mrs M Messman for her excellent technical assistance.

### REFERENCES

Anker P, Stroun M and Maurice PA (1975) Spontaneous release of DNA by human blood lymphocytes in vitro. *Cancer Res* **35**: 2375–2382

Anker P, Lefort F, Vasioukhin V, Lyautey J, Lederrey C, Chen XQ, Stroun M, Mulcahy HE and Farthing MJG (1997) K-*ras* mutations are found in DNA extracted from the plasma of patients with colorectal cancer. *Gastroenterology* **112**: 1114–1120

Bird AP (1986) CpG-rich islands and the function of DNA methylation. *Nature* **321**: 209–213

Chen XQ, Stroun M, Magnenat J-L, Nicod LP, Kurt A-M, Lyautey J, Lederrey C and Anker P (1996) Microsatellite alterations in plasma DNA of small cell lung cancer patients. *Nat Med* **2**: 1033–1035

Foster SA, Wong DJ, Barret MT and Galloway DA (1998) Inactivation of p16 in human mammary epithelial cells by CpG island methylation. *Mol Cell Biol* **18**: 1793–1801

Gardiner-Garden M and Frommer M (1987) CpG islands in vertebrate genomes. *J Mol Biol* **196**: 261–282

Goessl C, Heicappell R, Münker R, Anker P, Stroun M, Krause H, Müller M and Miller K (1998) Microsatellite analysis of plasma DNA from patients with clear cell renal carcinoma. *Cancer Res* **58**: 4728–4732

Gonzalez-Zulueta M, Bender CM, Yang AS, Nguyen T, Beart RW, Van Tornout JM and Jones PA (1995) Methylation of the 5′ CpG island of the p16/CDKN2 tumor suppressor gene in normal and transformed human tissues correlates with gene silencing. *Cancer Res* **55**: 4531–4535

Herman JG, Latif F, Weng W, Lerman MI, Zbar B, Liu S, Samid D, Duan D-S, Gnarra JR, Linehan WM and Baylin SB (1994) Silencing of the VHL tumor suppressor gene by DNA methylation in renal carcinoma. *Proc Natl Acad Sci USA* **91**: 9700–9704

Herman JG, Merlo A, Mao L, Lapidus RG, Issa J-PJ, Davidson NE, Sidransky D and Baylin SB (1995) Inactivation of the CDKN2/p16/MTS1 gene is frequently associated with aberrant DNA methylation in all common human cancers. *Cancer Res* **55**: 4525–4530

Hibi K, Robinson R, Booker S, Wu L, Hamilton SR, Sidransky D and Jen J (1998) Molecular detection of genetic alterations in the serum of colorectal cancer patients. *Cancer Res* **58**: 1405–1407

Issa J-PJ, Ottaviano YL, Celano P, Hamilton SR, Davidson NE and Baylin SB (1994) Methylation of the estrogen receptor CpG island links aging and neoplasia in human colon. *Nat Genet* **7**: 536–540

Jones PA and Buckley JD The role of the DNA methylation in cancer. *Adv Cancer Res* **54**: 1–23

Kamb A, Gruis NA, Weaver-Feldhaus J, Liu Q, Harshman K, Tavtigian SV, Stocker E, Day RS III, Johnson BE and Skolnick MH (1994) A cell cycle regulator potentially involved in genesis of many tumor types. *Science* **264**: 436–440

Laird PW and Jaenisch R (1994) DNA methylation and cancer. *Hum Mol Genet* **3**: 1487–1495

Leon SA, Shapiro B, Sklaroff DM and Yaros MJ (1977) Free DNA in the serum of cancer patients and the effect of therapy. *Cancer Res* **37**: 646–650

Lo YMD, Corbetta N, Chamberlain PF, Rai V, Sargent IL, Redman CWG and Wainscoat JS (1997) Presence of fetal DNA in maternal plasma and serum. *Lancet* **350**: 485–487

Merlo A, Herman JG, Mao L, Lee DJ, Gabrielson E, Burger PC, Baylin SB and Sidransky D (1995) 5′ CpG island methylation is associated with transcriptional silencing of the tumour suppressor p16/CDKN2/MYS1 in human cancers. *Nature Med* **1**: 686–692

Mulcahy HE, Lyautey J, Lederry C, Chen Q-X, Anker P, Alstead EM, Ballinger A, Farthing MJ and Stroun M (1998) A prospective study of K-*ras* mutations in the plasma of pancreatic cancer patients. *Clin Cancer Res* **4**: 271–275

Nawroz H, Koch W, Anker P, Stroun M and Sidransky D (1996) Microsatellite alterations in serum DNA of head and neck cancer patients. *Nat Med* **2**: 1035–1037

Nobori T, Miura K, Wu DJ, Lois A, Takabayashi K and Carson DA (1994) Deletions of the cycle-dependent kinase-4 inhibitor gene in multiple human cancers. *Nature* **368**: 753–756

Okamoto A, Demetrick DJ, Spillare EA, Hagiwara K, Hussain SP, Bennett WP, Forrester K, Gerwin B, Serrano M, Beach DH and Harris CC (1994) Mutations and altered expression of p16$^{INK4a}$ in human cancer. *Proc Natl Acad Sci USA* **91**: 11045–11049

Oto M, Miyake S and Yuasa Y (1993) Optimization of nonradioisotopic single strand conformation polymorphism analysis with a conventional minislab gel electrophoresis apparatus. *Ann Biochem* **213**: 19–22

Sanchez-Cespedes M, Monzo M, Rosell R, Pifarre A, Calvo R, Lopez-Cabrerizo MP and Astudillo J (1998) Detection of chromosome 3p alterations in serum DNA of non-small-cell lung cancer patients. *Ann Oncol* **9**: 113–116

Serrano M, Hannon GJ and Beach D (1993) A new regulatory motif in cell-cycle control causing specific inhibitions of cyclin D/CDK4. *Nature* **366**: 704–707

Serrano M (1997) The tumor suppressor protein p16$^{INK4a}$. *Exp Cell Res* **237**: 7–13

Shapiro B, Chakrabarty M, Cohn EM and Leon SA Determination of circulating DNA levels in patients with benign or malignant gastrointestinal disease. *Cancer* **51**: 2116–2120

Singer-Sam J, Yang TP, Mori N, Tanguay RL, Le Bon JM, Flores JC and Riggs AD (1990) DNA methylation in the 5′ region of the mouse PGK-1 gene and a quantitative PCR assay for methylation. In: *Nucleic Acid Methylation,* Vol. 128, Clawson GA, Willis DB, Weissbach A and Jones PA (eds), pp. 285–289. New York: Alan R. Liss

Stroun M, Anker P, Lyautey J, Lederrey C and Maurice PA (1987) Isolation and characterization of DNA from the plasma of cancer patients. *Eur J Cancer Clin Oncol* **23**: 707–712

Stroun M, Anker P, Maurice P, Lyautey J, Lederrey C and Beljanski M (1989) Neoplastic characteristics of the DNA found in the plasma of cancer patients. *Oncology* **46**: 318–322

Vasioukhin V, Anker P, Maurice P, Lyautey J, Lederrey C and Stroun M (1994) Point mutations of the N-*ras* gene in the blood plasma DNA of patients with myelodysplastic syndrome or acute myelogenous leukaemia. *Br J Haematol* **86**: 774–779

 © 1999 Cancer Research Campaign
